# Effective treatment of steroid and therapy-refractory acute graft-versus-host disease with a novel mesenchymal stromal cell product (MSC-FFM)

**DOI:** 10.1038/s41409-018-0102-z

**Published:** 2018-01-29

**Authors:** Peter Bader, Zyrafete Kuçi, Shahrzad Bakhtiar, Oliver Basu, Gesine Bug, Michael Dennis, Johann Greil, Aniko Barta, Krisztián M. Kállay, Peter Lang, Giovanna Lucchini, Raj Pol, Ansgar Schulz, Karl-Walter Sykora, Irene von Luettichau, Grit Herter-Sprie, Mohammad Ashab Uddin, Phil Jenkin, Abdulrahman Alsultan, Jochen Buechner, Jerry Stein, Agnes Kelemen, Andrea Jarisch, Jan Soerensen, Emilia Salzmann-Manrique, Martin Hutter, Richard Schäfer, Erhard Seifried, Thomas Klingebiel, Halvard Bonig, Selim Kuçi

**Affiliations:** 1 Department for Children and Adolescents, Division for Stem Cell Transplantation and Immunology, University Hospital Frankfurt, Frankfurt am Main, Germany; 2 University Children’s Hospital, Essen, Germany; 3 Department of Medicine II, University Hospital Frankfurt, Frankfurt, Germany; 4 Department of Haematology, Christie Hospital, Manchester, United Kingdom; 5 University Children’s Hospital Heidelberg, Heidelberg, Germany; 6 Department for Haematology and SCT, St. István and St. László Hospital, Budapest, Hungary; 7 University Children’s Hospital Tübingen, Tübingen, Germany; 8 Department of Hematology/Oncology, Great Ormond Street Hospital, London, United Kingdom; 9 Department of Haematology, University of Sheffield, Sheffield, United Kingdom; 10 University Children’s Hospital, Ulm, Germany; 11 Children’s Hospital, Medizinische Hochschule, Hannover, Germany; 12 Division of Pediatric Hematology/Oncology, Department of Pediatrics, Kinderklinik München Schwabing, Klinikum Rechts der Isar, Technische Universität München, München, Germany; 13 Department I for Internal Medicine, University Hospital Cologne, Cologne, Germany; 14 Department for Stem Cells & Immunotherapies, NHSBT, Birmingham, Great Britain UK; 15 Department of Pediatric Hematology/Oncology, King Abdullah Specialist Children’s Hospital, Riyadh, Saudi Arabia; 16 Oslo University Hospital Rikshospitalet, Department of Pediatric Medicine, Section for Pediatric Hematology/Oncology, Oslo, Norway; 17 Department for Hemato-Oncology, Schneider Children’s Medical Center of Israel, Petach Tikva, Israel; 18 B-A-Z County Hospital, Pediatric Haematology and Stem Cell Transplantation Unit, Miskolc, Hungary; 19 German Red Cross Blood Center Frankfurt and Institute of Transfusion Medicine and Immunohematology, Goethe University Medical Center, Frankfurt am Main, Germany

## Abstract

The inability to generate mesenchymal stromal cells (MSCs) of consistent potency likely is responsible for inconsistent clinical outcomes of patients with aGvHD receiving MSC products. We developed a novel MSC manufacturing protocol characterized by high in vitro potency and near-identity of individual doses, referred to as “MSC-Frankfurt am Main (MSC-FFM)”. Herein, we report outcomes of the 69 patients who have received MSC-FFM. These were 51 children and 18 adults with refractory aGvHD grade II (4%), III (36%) or IV (59%). Patients were refractory either to frontline therapy (steroids) (29%) or to steroids and 1–5 additional lines of immunosuppressants (71%) were given infusions in four weekly intervals. The day 28 overall response rate was 83%; at the last follow-up, 61% and 25% of patients were in complete or partial remission. The median follow-up was 8.1 months. Six-month estimate for cumulative incidence of non-relapse mortality was 27% (range, 16–38); leukemia relapse mortality was 2% (range, 0–5). This was associated with a superior six-month overall survival (OS) probability rate of 71% (range, 61–83), compared to the outcome of patients not treated with MSC-FFM. This novel product was effective in children and adults, suggesting that MSC-FFM represents a promising therapy for steroid refractory aGvHD.

## Introduction

Acute graft-versus-host disease (aGvHD) remains a major complication and cause of mortality after allogeneic hematopoietic stem cell transplantation (HSCT). Despite a calcineurin inhibitor-based GvHD prophylaxis without in vivo T cell depletion, approximately 40% of patients remain at risk for developing GvHD. The first-line GvHD therapy continues to be corticosteroids, to which about half of the patients respond within a few days [1–3]. Despite administration of additional lines and apparently irrespective of the selected therapeutic agent, those patients who are refractory to steroids have very poor outcomes with overall survival (OS) as low as 20%. Third-line treatments include mycophenolate mofetil (MMF), T-cell depleting and anti-cytokine antibodies and, most recently, Jak inhibitors [4]. Both the aGvHD itself and adverse effects of GvHD treatment, such as hepatic and renal toxicity, opportunistic infections, relapse of the underlying malignant disease and secondary graft failure, contribute to patients’ death [1, 2]. The first promising alternative to immunosuppressants dates back to 2004, when Le Blanc et al. [5]. reported in a landmark paper resolution of a treatment-refractory grade IV aGvHD in a 9-year-old boy by infusion of bone marrow-derived mesenchymal stromal cells (MSCs) isolated from the mother. This prompted an initial trial in which eight patients with grade III–IV, biopsy-proven steroid-refractory GVHD were infused with MSCs, leading to clinical improvement in six [6]. Based on the encouraging results of these two initial reports, a first phase II trial in 55 adult and pediatric patients with steroid-refractory acute grades II–IV GvHD across 5 European centers was conducted. Bone marrow-derived MSC infusions induced a complete response (CR) in 30 patients and partial response (PR) in 9, with 16 non-responders (NR) to MSC treatment. No side effects related to MSC infusions and no differences in response relative to MSC donor MHC matching were reported [7].

The majority of pilot and phase I/II studies confirmed the safety and efficacy of MSC infusions in the aGvHD setting in both pediatric and adult patient populations, although with variable results [8–11]. All clinical studies demonstrated a trend towards a better clinical response in children compared with adults [12, 13].

Differences in the results of clinical studies may be due to highly variable quality of MSCs used in the various trials, and more specifically, the lack of a robust manufacturing process which could generate sufficient doses of MSCs with batch-to-batch consistency. We recently reported a novel method for MSC generation from pooled bone marrow-derived mononuclear cells of multiple allogeneic donors [14]. A national marketing authorization based on the “hospital exemption” clause of the European advanced therapy medicinal product (ATMP) guidelines was obtained for this MSC product, termed MSC-Frankfurt am Main (MSC-FFM). Herein, we report the outcomes of the first 69 patients with steroid-resistant or treatment-refractory aGvHD treated with MSC-FFM in a routine clinical setting in allogeneic transplant centers across six countries.

## Subjects and methods

### Patients and GvHD scoring

Children and adults with steroid-refractory aGvHD (lack of steroid responsiveness for at least 5 days) or treatment-resistant aGvHD (refractoriness to steroids and at least one additional line of immunosuppressive therapy) after allogeneic HSCT irrespective of HLA matching between patient and donor and GvHD prophylaxis were eligible to receive MSC-FFM. In order to receive MSC, patient and transplant characteristics as well as staging and prior treatment of aGVHD had to be submitted to one of us (PB). Parents or patients gave their informed consent. Primarily, GvHD was diagnosed clinically; histological or other non-clinical evidence was only sought to rule out alternative diagnoses in unclear cases. Acute GvHD scoring was performed using the Seattle-Glucksberg modified criteria [15, 16]. MSC-FFM was dosed at 1–2 × 10^6^/kg body weight as a once-weekly rapid intravenous infusion for 1–4 successive weeks. Response was defined as either CR in patients who showed complete resolution of all signs of aGvHD, PR in patients who showed GvHD reduction by at least one grade according to the Glucksberg criteria, or non-response (NR) at day 28 after first MSC transfusion. Initially, only 26 children were treated with MSC-FFM as recently reported [14]. Consecutively, more patients (adults and children) with severe steroid and treatment-refractory aGvHD received these MSC products. Herein, we report 69 patients with refractory aGvHD who were treated with MSC-FFM in 14 allogeneic transplant services based in six countries (Germany, Hungary, Israel, Norway, Saudi-Arabia, and UK). Details on the patients’ characteristics are presented on Table 1. Of these 69 patients, 26 children were already reported in the initial description of the MSC manufacturing protocol [14].

**Table 1:** Characteristics of patients

	*N* *= *69	100%
Sex		
Female	21	(30%)
Male	48	(70%)
Age at HSCT		
≤18 y	51	(74%)
Median (range) y	8.2	(0.5–18.0)
>18 y	18	(26%)
Median (range) y	45.5	(18.9–65.6)
Diagnosis		
Malignant	51	(74%)
Non-malignant	18	(26%)
Donor		
MSD	14	(20%)
MUD	44	(64%)
Haploidentical FD	11	(16%)
Source		
BM	36	(52%)
PBSC	32	(46%)
CB	1	(1%)
Conditioning regimen		
TBI + others	15	(22%)
BU + others	15	(22%)
TREO + others	21	(30%)
Others	18	(26%)
In vivo T cell depletion for conditioning		
Without	17	(25%)
ATG	34	(49%)
Campath	14	(20%)
Others	4	(6%)
GVHD Prophylaxis		
Without	10	(14%)
CSA alone	11	(16%)
CSA + MTX	26	(38%)
CSA + MMF	7	(10%)
Siro + Tacrolimus	4	(6%)
MMF + Tacrolimus	4	(6%)
Others	7	(10%)

Data are *n* (%) or median (range) for age

*HSCT* hematopoietic stem cell transplantation, *y* years, *MSD* matched sibling donor, *MUD* matched unrelated donor (>9/10; high resolution match), *FD* family donor, *BM* bone marrow, *PBSC* peripheral blood stem cell, *CB* cord blood, *TBI* total body irradiation, *BU* busulfan, *TREO* treosulfan, *ATG* antithymocyte globulin, *GVHD* graft-versus-host disease, *CSA* cyclosporin A, *MTX* methotrexate, *MMF* mycophenolate mofetil

### MSC-FFM

The development of MSC-FFM manufacturing protocol was previously reported [14]. Donors were selected in accordance with national (German Transfusion Act and ancillary legislation) and international (FACT/JACIE and WMDA) regulation [17]. Pooled mononuclear cells from bone marrow (BM-MNCs) of eight donors were cultured in platelet lysate-supplemented media in order to generate MSCs. Generated MSCs were then frozen in >200 aliquots (MSC bank) and were further used to generate equipotent clinical-grade MSC-FFM batches in various sizes for patients with different weights.

### Statistical analysis

The response rates (OR, NR) per categories were compared using Fisher’s exact test excluding these patients (*N* = 2) from whom no day 28 report was available. We estimated the median survival follow-up time since first MSC infusion using the reverse Kaplan–Meier method.

The OS probability was estimated using Kaplan–Meier statistics. The survival time was considered from the date of the first MSC infusion to the death date or the last follow-up (LFU) date for censored patients. The log-rank test was used to estimate the significance between OS. The non-relapse mortality (NRM) was defined as death from any cause without previous relapse or progression. Cumulative incidence curves were used to estimate the NRM considering relapse mortality (RM) as a competing risk [18]. Gray’s test was used to compare the statistical significance of the difference between the cumulative incidences [19]. The results are expressed as probability or cumulative incidences with its 95% confidence interval. The six-month predicted estimates for OS and cumulative incidences were considered in accordance with other studies [20–22]. All tests were two-tailed, and a *P*-value of <0.05 was considered to be statistically significant. Statistical analyses were performed using the statistical software R, version 3.3.3 (R Project for statistical computing, www.r-project.org/).

## Results

### Safety and tolerability

Sixty-nine patients received a total of 212 doses of MSC-FFM. MSC-FFM was administered intravenously immediately after thawing, i.e., while still ice-cold, as a short infusion over no less than 10 min with clinical and vital parameter monitoring. The maximum volume of product and maximum dose of DMSO were 3–4 ml/kg and 0.3–0.4 g/kg, respectively, in the smallest three children weighing between 12–14 kg, and lower than that in all other patients, thus significantly below reported toxic doses of DMSO [23]. Accordingly, there was only one case each of nausea/vomiting, presumably due to DMSO, and headache, both in children, presumably due to the cold infusion solution. No other adverse effects were reported. Thus far, no limiting acute toxicity has been associated with MSC-FFM infusions. Long-term adverse events of particular interest include relapse of the underlying disease or severe infections. Since classical immunosuppressants non-specifically suppress alloreactivity, adaptive and graft-versus-leukemia responses alike, approximately half of the deaths in GvHD patients are due to infection and leukemic relapse which must be considered sequelae of GvHD treatment, as opposed to GvHD itself. With MSC-FFM given on top of immunosuppressive drugs the six month predicted relapse of the underlying mortality rate was only 2% (95% CI 0–5) and the total non-relapse mortality only 27% (16–38) (Table 2). Given the small number of events, overall cohort size, and our inability to distinguish between adverse effects of the classical immunosuppressants and the added effect of MSC-FFM, current data suggest that our cell-based product does not induce long-term adverse effects.

**Table 2:** Cause of death related to the day 28 response

	Severity of aGvHD prior to MSC-FFM	Treatment prior to MSC-FFM	CR (*N* = 22)	PR (*N* = 35)	NR (*N* = 10)	No report (*N* = 2)	Total
TRM							
Aspergillosis/candida	III/IV	SR/SR	2				2
Mucor	III/IV	SR/TR, 4	1	1			2
Sepsis	III/IV	SR/TR, 3	1		1		2
Virus/Adenovirus	IV/IV	TR, 5/TR, 4		2			2
Cerebral haemorrague	IV	TR, 6	1				1
GvHD	IV/IV/IV/IV	TR, 5/TR, 3/TR, 5/TR, 3		2	1	1	4
MOF	III/IV	TR, 5/ST			2		2
Acute abdomen due to strangulated hernia	III	ST	1				1
No data	IV	TR, 5			1		1
Thrombembolism + HSV pneumonia	IV	TR, 3				1	1
Relapse of the underlying disease	II/III/III	TR, 3/TR, 4/TR, 3		3			3
Total			6 (27%)	8 (23%)	5 (50%)	2 (100%)	21 (30%)

Numbers after TR indicates the number of therapy lines used before the treatment with MSC-FFM

*aGvHD* acute graft-versus-host disease, *CR* complete response, *PR* partial response, *NR* non-response, *TRM* treatment related mortality, *SR* steroid refractory, *TR* treatment refractory, *GvHD* graft-versus-host disease, *MOF* multiple organ failure, *HSV* herpes simplex virus, *MSC-FFM* Mesenchymal Stromal Cell-Frankfurt am Main

### Response and clinical efficacy of MSC-FFM

**Fig. 1:**
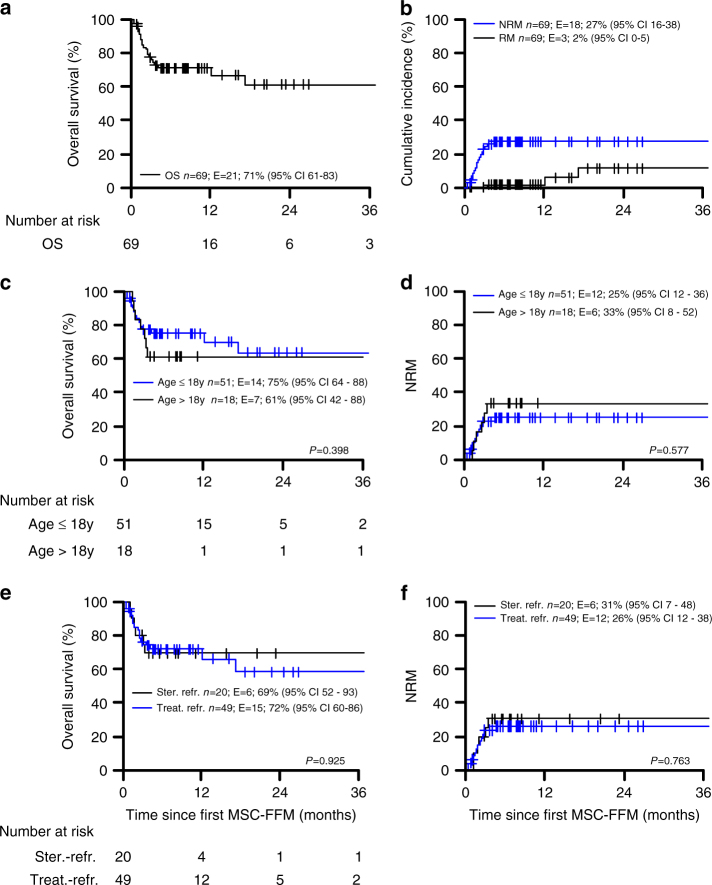
Overall survival and cumulative incidences with estimates at six months after first MSC-FFM administration. **a** The overall survival (OS) of all patients treated with MSC-FFM. **b** The cumulative incidence for all patients as to non-relapse mortality (NRM) and relapse mortality (RM) by the underlying disease is displayed. **c**, **d** Overall survival and non-relapse mortality related to the age and therapy prior to MSC-FFM administration (**e**, **f**). (Colour figure online)

At day 28, 22 (32%) patients achieved CR, 35 (51%) PR, 10 (14%) NR and for two of patients (3%) there were no day 28 data available. This resulted in an overall response (OR) of 83%. At the LFU (median follow-up: 8.19 months; range, 0.9–54.02 months), 42 (61%) patients were in CR, 17 (25%) patients in PR, and 10 patients (14%) were NR. These response rates resulted in a predicted six month non-relapse mortality rate (NRM) of 27% (95% CI 16–38) and cumulative leukemia relapse mortality incidence of 2% (0–5), for an OS rate of 71% (61–83) (Fig. 1a, b; Table 3).

**Table 3:** Response status at day 28 after first administration of MSC-FFM and at last follow-up

	*N*	(%)	Day 28										*P*	Last follow-up								*P*
			CR		PR		NR		no report		OR			CR		PR		NR		OR		
			*N*	(%)	*N*	(%)	*N*	(%)	*N*	(%)	*N*	(%)		*N*	(%)	*N*	(%)	*N*	(%)	*N*	(%)	
All	69	(100%)	22	(32%)	35	(51%)	10	(14%)	2	(3%)	57	(83%)		42	(61%)	17	(25%)	10	(14%)	59	(86%)	
Age													0.717									1
≤18 y	51	(74%)	13	(25%)	28	(55%)	8	(16%)	2	(4%)	41	(80%)		33	(65%)	10	(20%)	8	(16%)	43	(84%)	
>18 y	18	(26%)	9	(50%)	7	(39%)	2	(11%)	—	—	16	(89%)		9	(50%)	7	(39%)	2	(11%)	16	(89%)	
Diagnosis													0.706									0.436
Malignant	51	(74%)	17	(33%)	26	(51%)	7	(14%)	1	(2%)	43	(84%)		31	(61%)	14	(27%)	6	(12%)	45	(88%)	
Nonmalignant	18	(26%)	5	(28%)	9	(50%)	3	(17%)	1	(6%)	14	(78%)		11	(61%)	3	(17%)	4	(22%)	14	(78%)	
Severity of aGVHD prior to MSC-FFM													0.389									0.472
Grade II	3	(4%)	1	(33%)	2	(67%)	—	—	—	—	3	(100%)		2	(67%)	1	(33%)	—	—	3	(100%)	
Grade III	25	(36%)	11	(44%)	12	(48%)	2	(8%)	—	—	23	*(92%)*		15	(60%)	8	(32%)	2	(8%)	23	(92%)	
Grade IV	41	(59%)	10	(24%)	21	(51%)	8	(20%)	2	(5%)	31	(76%)		25	(61%)	8	(20%)	8	(20%)	33	(80%)	
Therapy prior to MSC-FFM													0.655									0.053
Steroid-refractory	20	(29%)	13	(65%)	6	(30%)	1	(5%)	—	—	19	(95%)		16	(80%)	4	(20%)	—	—	20	(100%)	
Treatment-refractory	49	(71%)	9	(18%)	29	(59%)	9	(18%)	2	(4%)	38	(78%)		26	(53%)	13	(27%)	10	(20%)	39	(80%)	

Data are *n* (%). Table shows the reported response status. Comparison between response status (OR, NR) per patient characteristic was performed using Fisher’s exact test

*MSC-FFM* Mesenchymal Stromal Cell-Frankfurt am Main, *CR* complete response, *PR* partial response, *NR* non-response, *OR* overall response, *y* years, *aGVHD* acute graft-versus-host disease

**Fig. 2:**
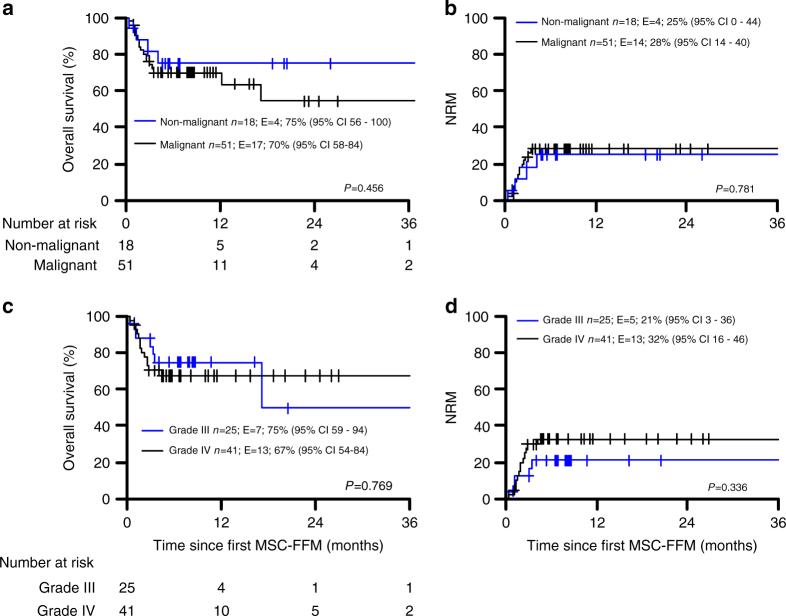
Overall survival and cumulative incidences with probabilities and estimates at 6-months after first MSC-FFM administration according to disease (**a**,** b**), and (**c**, **d**) severity of aGvHD prior treatment with MSC-MMF. In this panel patients with aGvHD grade II (*n* = 3, 4%) are not shown. (Colour figure online)

Patients with aGvHD grade III or grade IV had at 6 month an estimated OS probability of 75% (59–94) and 67% (54–84), respectively, which seems to be superior to historically expected survival rates for patients with such severe aGvHD (Fig. 2c, d; Table 3).

Clinical responsiveness did not differ between children (<18 years, *n* = 51) and adults (>18 years, *n* = 18): Of the 51 children, 13 (25%) and 28 (55%) reached CR or PR by day 28, respectively. Eight (16%) were NR and in 2 (4%) patients no day 28 report was available. Among those 18 patients >18 years of age, 9 (50%) achieved CR, 7 (39%) PR and 2 (11%) patients did not respond. This resulted in an OR of 80% in children and 89% in adults (Table 3). Thus estimated six-month survival rates in children and adults were 75% (64–88) and 61% (42–88), respectively (*P* = 0.398). Similarly, non-relapse mortality at six-month was 25% (12–36) or 33% (8–52) in children vs. adults, respectively (*P = *0.577) (Fig. 1c, d).

### Steroid refractory and treatment refractory patients

The earliest studies used steroid refractoriness for 5–7 days as indication for MSC treatment. We therefore also stratified our patients with respect to whether the patients were refractory to high-dose steroids only vs. therapy-refractory, i.e., having received and failed to respond to additional lines of treatment on top of high-dose steroids. Only 20 (29%) patients belonged to the group of steroid-refractory patients. Of these, 19 (95%) patients responded to MSC-FFM, 13 (65%) achieved CR, 6 (30%) PR and 1 (5%) patient did not respond by day 28. At the LFU (median follow-up: 8.19 months; range: 0.9–54.02 months) 16 patients (80%) had achieved CR and 4 PR (20%). The majority of the patients (*N* *= *49, 71%) were treatment-refractory to at least three or more lines of immune suppressive treatment. Of these, 38 (78%) patients responded, 9 (18%) with CR, 29 (59%) with PR, and 9 (18%) showed no response at day 28; in 2 (4%) patients no day 28 report was available. At the LFU, MSC-FFM treatment resulted in an OR in 39 (80%), 26 (53%) achieved CR, 13 (27%) patients PR, and 10 (20%) patients did not respond (Table 2). The outcomes of steroid-refractory vs. treatment-refractory patients did not reveal statistically significant differences. The predicted six-month OS was 69% (52–93) vs. 72% (60–86) for steroid-refractory vs. treatment-refractory patients (*P = *0.925) with a NRM of 31% (7–48) vs. 26% (12–38) (*P = *0.763) (Fig. 1e, f).

**Fig. 3:**
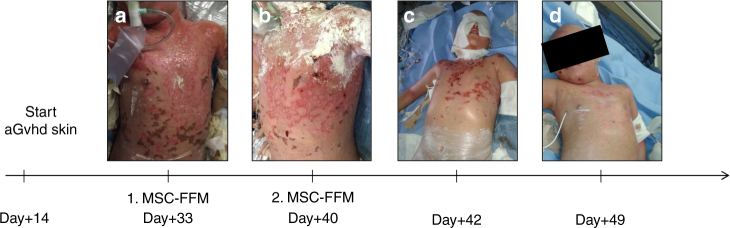
Skin GvHD responds to MSC-FFM. **a** A representative patient with severe cutaneous aGvHD is shown at day +33, when the first dose of MSC-FFM was given. **b** Improved skin at day +40 when a second dose of MSC-FFM was infused. **c**, **d** show continuously improved skin until day +49 when all involved areas completely responded

**Fig. 4:**
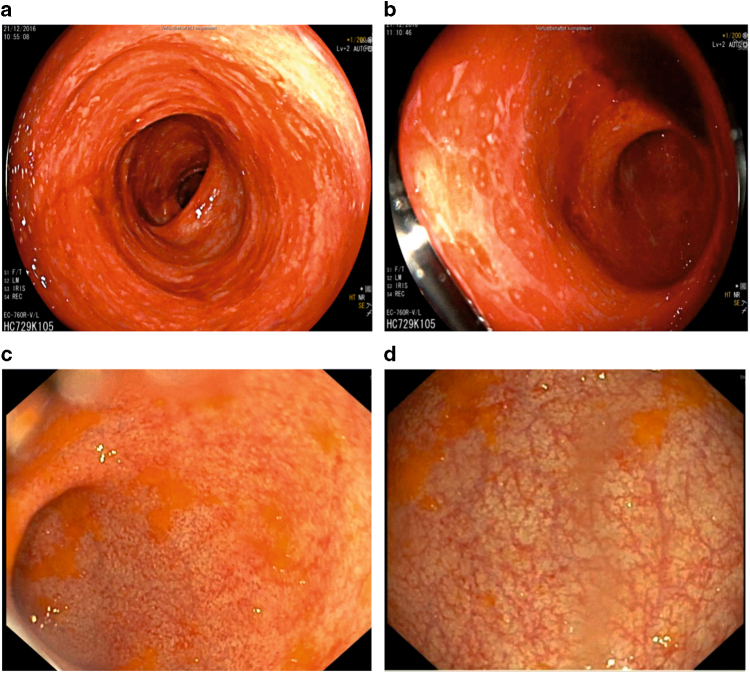
Intestinal GvHD responds to MSC-FFM. **a**, **b** Colonoscopy results two days before first MSC-FFM infusion. Severe GvHD with multiple ulcera and spontaneous bleeding was confirmed by histology. **c**, **d** Resolution of intestinal GvHD showed only mild proctitis two weeks after the first MSC-FFM application

There was also no difference in either six-month OS or in NRM of patients with non-malignant (*N = *18) compared to patients with malignant disease (*N = *51) (Fig. 2a, b). Moreover, efficacy of MSC-FFM was not different in the treatment of children (≤18 years, *n* = 51) compared to adult patients (>18 years, *N = *18), neither in OS nor NRM (Fig. 1c, d). This effect was observed in most of the patients with severe skin (Fig. 3), or intestinal GvHD (Fig. 4), who showed impressive responses to MSC-FMM. A six-year old patient with ALL developed an aGvHD of the skin at day +14, which was unresponsive to either steroids, MMF or basiliximab. The patient received MSC-FFM at day +33 (Fig. 3a, permission to publish picture obtained) and the skin improved substantially 3 days later. Seven days after the first MSC-MMF infusion (Fig. 3b) skin improved continuously (Fig. 3c) and the patient received a second dose. Sixteen days after the first MSC-FFM infusion (day 49), the aGvHD showed a complete response (Fig. 3d).

In addition, MSC-FFM was very effective in the treatment of aGvHD in adult patients. In a 23 year old male patient with AML, GvHD prophylaxis was discontinued because of decreasing donor chimerism on day +115. Three days later, the patient developed aGvHD grade III (skin stage 2, gut stage 3) (Fig. 4a, b). Acute GvHD proofed to be refractory to steroids and tacrolimus. The patient received altogether 3 doses of MSC-FFM on days +139, +146, and +158. Clinical improvement started already 3 days after the first MSC-FFM application and 14 days thereafter, the colonoscopy showed only mild proctitis (Fig. 4c, d).

## Discussion

Mesenchymal stromal cells are one of the more recent therapeutic modalities considered for aGvHD treatment. Despite the general consensus that MSCs appear to be well-tolerated (safe) and effective for the treatment of various diseases, there has been no unambiguous evidence in the field favoring MSC treatment due to inconsistencies in the outcome of GvHD clinical trials [5, 7–9, 14]. Here, we report one of the largest cohorts of patients with refractory aGvHD who, most notably, received the same, standardized MSC-FFM therapy. What distinguishes this report from many of the previous reports is the sheer size of the cohort and its multi-national and multi-hospital routine post-approval setting. With three exceptions, all patients were suffering from grade III (36%) or IV (59%) GvHD at the time of MSC-FFM therapy. This is a more severely ill cohort than in most published series, as well as it is the most heavily pre-treated one, since only 29% of the patients were steroid-refractory, while the remainder had received as many as six additional lines of treatment before the decision was made to treat them with MSC-FFM. Of significant interest for the definition of outcome measures for future aGvHD trials, while many of the PR improved to CR over the course of the observation period, very few of the day 28 non-responders had delayed responses, most remained non-responders throughout.

This observation indicates the predictive value of day 28 responses for overall therapeutic benefit and suggests its use as a surrogate outcome parameter, in agreement with published work [24]. Accordingly, in this study we also used day 28 response as an outcome parameter. In our cohort, 57 of 69 patients (83%) responded to the MSC treatment by day 28 (OR), which is much more encouraging than the results from a randomized placebo-controlled Prochymal aGvHD study. Extraction of data from the Prochymal 280 study from the Australian regulatory agency indicates that the trial enrolled 173 aGvHD patients with steroid refractoriness, 28 of which children; with only 73% grade C and grade D. Considering, therefore, only the response data for the 126 patients with severe GvHD, a response rate of 30.2% was reported. OS at six months for the entire MSC-treated cohort was 34%, compared to 42% for the placebo group. Thus, this does not provide convincing evidence to support efficacy of Prochymal in adults with aGvHD, while benefit in children was suggested by sub-group analyses within the 280 trial and supported by the 275 trial. Although the primary end point in that study was not achieved for the whole group of patients, there was a significant benefit over placebo group if the liver and GI tract were affected [25].

Le Blanc et al. reported similar response rates to the MSC-treatment of 25 children and 30 adults with acute GvHD compared to the Prochymal data in a multicenter phase II study [7]. The OR for their cohort of patients was 70.5% at median time from 18 days (3–63 days) and not at day 28 as an outcome end point used in the current study. Response rate in our cohort of patients is also superior to that reported by Introna et al. [26] in a cohort of 15 pediatric patients; that study demonstrated an OR of 66.7% compared to our pediatric cohort (80%). However, in their patient cohort, only 25% of the patients exhibited aGvHD over grade III, whereas in our series, 96% of the patients exhibited aGvHD grade III or IV. Lucchini et al. [8] observed a 62.5% OR among eight patients with aGvHD (50% grade I/II and 50% grade III/IV). Similar findings were reported by Prasad et al. [27]. In their compassionate use study the authors could show an OR of 66.7% at day 32 in 12 pediatric patients. Kurtzberg et al. [24] achieved a 61.3% OR rate in a large cohort of 75 pediatric patients after treatment of aGvHD with Prochymal. In contrast, we demonstrate an excellent response rate not only in children but also in adult patients. The fact that we obtained these results with a highly challenging patient cohort (96% grade III/IV and only 4% grade II) suggests the advantage of the treatment of aGvHD with MSC-FFM. We attribute this superiority to the MSC preparation procedure. Cultivating the cells out of a pool of mononuclear cells from eight different donors led to a higher in vitro suppression of a mixed lymphocyte reaction [14]. Moreover, as all therapeutic doses of the MSC-FFM product have exactly the same, standardized potential, every patient has received the same product and not as in forgoing trials different MSCs preparations from different donors. All this makes the treatment with MSC-FFM unique and distinguishes the treatment from all other reports so far.

Salmenniemi and colleagues [28] reported an OR at day 28 for adults and children of 50% and 88%, respectively. The survival in the adult cohort was disappointingly low with approximately 30% at one year, while one-year survival of the MSC-treated children was 100%.

Recently, Dotoli et al. [29] and von Dalowski et al. [30] reported poor outcomes in adult GvHD patients treated with MSCs after steroid-refractoriness (1-year OS: 19.6% and 19%, respectively). Likewise, Salmenniemi et al. reported OS of 22% for their MSC-treated patients with GvHD after a median follow-up of 767 days (range 74–1270 days) from diagnosis [28]. Remarkably, in our cohort there was no significant difference in OS between the treated children/adolescents with MSCs (75% (95% CI 64–88)) and adults (61% (42–88)) (*P = *0.398). While too small a cohort to derive statistically relevant information, these data are supportive for the current license of MSC-FFM for both children and adults with refractory aGvHD. In addition, the OS in children of our cohort was also better than in all previous clinical studies as reported to date [7, 24, 27]. The OR rates for steroid-refractory patients at day 28 were 100 and 80% for treatment-resistant patients (s. Table 3) at LFU. Although this difference did not achieve statistical significance (*P* = 0.053) it implies that treatment with MSC-FFM should be started as early as possible. For the time being, we recommend to start treatment with MSC-FFM as early as definitions for steroid refractivity are met [1].

Noteworthy, the six month OS of patients with grade II aGvHD (*N = *3) was 100%, with grade III (*N* = 25) 75% (59–94) and with grade IV (*N = *41) was 67% (54–84), suggesting the best survival rate reported thus far and approaching those for patients without severe GvHD (Table 4) [7, 8, 24, 26, 27, 29, 30].

**Table 4:** Results of clinical studies using MSC for treatment of steroid refractory aGVHD patients

Publication		GVHD	Response at day +28	OS observation time	Predicted OS Mean [95% CI]
Lucchini et al. [7]	*N* = 11 children	aGVHD I-II: *N* = 4 (36%); III-IV: *N* = 4 (36%); cGVHD: *N* = 3 (27%)	CR = 23.8%; PR = 47.6%;OR = 71.4%	8 [4–18] mo	8/11 = 73%
*Only aGVHD	*N* = 8	aGVHD I-II: *N* = 4 (50%); III-IV: *N* = 4 (50%)	CR = 37.5%; PR = 25%; OR = 62.5%		5/8 = 62.5%
Prasad et al. [20]	*N* = 12 children	aGVHD III: *N* = 5 (42%); IV: *N* = 7 (58%)	CR = 17%; PR = 50%; OR = 67%	2-years OS	40% [20–82%]
Introna et al. [19]	*N* = 40				
	*N* = 15 children	aGVHD II: *N* = 9 (60%); III-IV: *N* = 3 (20%); cGVHD: *N* = 1 (7%); overlap: *N* = 2 (13%)	CR = 46.6%; PR = 20%; OR = 66.6%	1-year OS	66.7 ± 12.7%
	*N* = 25 adults	aGVHD II: *N* = 2 (8%); III-IV: *N* = 17 (68%); cGVHD: *N* = 2 (8%); overlap: *N* = 4 (16%)	CR = 16%; PR = 52%; OR = 68%	1-year OS	40.0 ± 9.8%
Le Blanc et al. [6]	*N* = 55	aGvHD II: *N* = 5 (9%); III: *N* = 25 (45.45%);	Median time = 18 (3–63) days CR = 54.5%; PR = 16%; OR = 70.5%	2-years-OS	35% [22–38%] (for all patients)
	*N* = 25 children	IV: *N* = 25 (45.45%)			45% [23–67%] (children)
	*N* = 30 adults				26% [10–42%] (adults)
Kurtzberg et al. [17]	*N* = 75 children	Grad B: *N* = 9 (12%); C: *N* = 21 (28%); D: *N* = 45 (60%)	OR = 61.3%	day + 100 for OS	57.3%
Dotoli et al. [22]	*N* = 46	aGVHD III: *N* = 10 (21.74%); IV: *N* = 36 (78.26%)	CR = 6.5%; PR = 43.5%; OR = 50%	2-years OS	17.4%
	*N* = 16 children				
	*N* = 30 adults				
Dalowski et al. [23]	*N* = 58 adults	aGVHD I: *N* = 1 (2%); II: *N* = 3 (5%); III: *N* = 8 (14%)	CR = 9%; PR = 38%; OR = 47%	1-year OS	19% [9–29%]
		IV: *N* = 46 (79%)		2-years OS	17% [7–26%]
Salmenniemi et al. [21]	*N* = 30		CR = 22%; VGPR = 17%; PR = 11%;	1-year OS	48%
	*N* = 8 children	aGVHD II: *N* = 1 (13%); III: *N* = 5 (63%); IV: *N* = 2 (25%)	NR = 50%	2-years OS	29%
	*N* = 22 adults	aGVHD II: *N* = 1 (6%); III: *N* = 9 (50%); IV: *N* = 8 (44%)			
Bader et al. 2017	*N* = 69	aGVHD II: *N* = 3 (4%); III: *N* = 25 (36%); IV: *N* = 41 (59%)	CR = 31.9%; PR = 50.7%; OR = 82.6%	6-mo OS	71 ± 6%
	*N* = 51 children				
	*N* = 18 adults				

*aGvHD* acute graft-versus-host disease, *MSC-FFM* Mesenchymal Stromal Cell-Frankfurt am Main, *GVHD* graft-versus-host disease, *OS* overall survival, *cGVHD* chronic graft-versus-host disease, *CR* complete response, *VGPR* very good partial response, *PR* partial response, *OR* overall response, *CI* confidence interval, *mo* months

In conclusion, MSC-FFM offers an excellent salvage therapy for both steroid and treatment-refractory aGvHD, warranting further clinical evaluation.
